# Correlation of Gleason Scores with Diffusion-Weighted Imaging Findings of Prostate Cancer

**DOI:** 10.1155/2012/374805

**Published:** 2011-12-15

**Authors:** Rajakumar Nagarajan, Daniel Margolis, Steven Raman, Ke Sheng, Christopher King, Robert Reiter, M. Albert Thomas

**Affiliations:** ^1^Department of Radiological Sciences, David Geffen School of Medicine, University of California, Los Angeles, CA 90095-1721, USA; ^2^Department of Radiation Oncology, David Geffen School of Medicine, University of California, Los Angeles, CA 90095-1721, USA; ^3^Department of Urology, David Geffen School of Medicine, University of California, Los Angeles, CA 90095-1721, USA

## Abstract

The purpose of our study was to compare the apparent diffusion coefficient (ADC) derived from diffusion-weighted imaging (DWI) of prostate cancer (PCa) patients with three classes of pathological Gleason scores (GS). Patients whose GS met these criteria (GS 3 + 3, GS 3 + 4, and GS 4 + 3) were included in this study. The DWI was performed using *b* values of 0, 50, and 400 s/mm^2^ in 44 patients using an endorectal coil on a 1.5T MRI scanner. The apparent diffusion coefficient (ADC) values were calculated from the DWI data of patients with three different Gleason scores. In patients with a high-grade Gleason score (4 + 3), the ADC values were lower in the peripheral gland tissue, pathologically determined as tumor compared to low grade (3 + 3 and 3 + 4). The mean and standard deviation of the ADC values for patients with GS 3 + 3, GS 3 + 4, and GS 4 + 3 were 1.135 ± 0.119, 0.976 ± 0.103 and 0.831 ± 0.087 mm^2^/sec. The ADC values were statistically significant (*P* < 0.05) between the three different scores with a trend of decreasing ADC values with increasing Gleason scores by one-way ANOVA method. This study shows that the DWI-derived ADC values may help differentiate aggressive from low-grade PCa.

## 1. Introduction

Prostate cancer (PCa) is the most common malignancy among men in the USA, with an estimated 217,730 new cases and 32,050 PCa-related deaths in 2010 [1]. The incidence of PCa increases with age, and it is very uncommon in men younger than 50 years old. With greater longevity and increased awareness of the disease leading to more men requesting screening, it is to be expected that there will be an increase in the number of patients diagnosed with PCa in the future. Most men diagnosed with PCa ultimately survive the disease and die of other causes. The overall 5-year survival rate is 99% for all stages, but only 34% when there are distant metastases [2]. The aim of PCa management is to identify, treat, and cure patients with aggressive disease that may prove fatal but to avoid overtreating those in whom the disease is unlikely to be life threatening. Most patients diagnosed with PCa have localized disease confined to the prostate. A small number with high-grade tumors will progress to develop local, extracapsular tumor extension and distant metastases.

Prostate tumors are graded according to their pathological appearance with a Gleason score (GS), which represents the sum of the dominant and subdominant histological patterns (grades). High GSs indicate aggressive tumors with increased potential for local and distant spread; Gleason grading has been shown to provide a spectrum of risk for all patients [3]. Magnetic resonance imaging (MRI) provides incremental value to clinical findings in staging patients of intermediate risk. For example, organ-confined disease implies that the patient may benefit from local therapy such as surgery [4]. MRI is more accurate than either digital rectal examination (DRE) or transrectal ultrasound (TRUS) biopsy in preoperative anatomical localization of PCa [5]. The sensitivity and specificity of T_2_-weighted imaging for PCa vary widely due to differences in imaging techniques, reference standards, criteria for defining disease involvement on MRI, and interobserver variability [6]. In a meta-analysis by Sonnad et al. T_2_-weighted imaging showed a maximum joint sensitivity and specificity rate of 74% for the staging of PCa [7]. In T_2_-weighted imaging, regions of PCa show decreased signal intensity relative to normal peripheral (PZ) tissue because of increased cell density and a loss of prostatic ducts [8]. This finding is nonspecific, however, because other diseases such as prostatitis or hyperplasia can also cause low signal intensity in T_2_-weighted imaging [9–12].

 Diffusion-weighted imaging (DWI) is another MR-based technique that probes functional characteristics of tissues. The clinical success of DWI has led to a broadening application in the prostate gland. Rapid changes in diffusion properties can be identified by calculating the apparent diffusion coefficient (ADC). Dickinson et al. [13] showed the standardizing multiparametric magnetic resonance imaging (mpMRI) for PCa detection, localization, and characterization. The use of DWI as a tool for the evaluation and management of prostatic cancer has grown steadily in the past decades [14–16]. The purpose of the study was to record DWI and to compare ADC values derived from DWI in PCa patients with three different Gleason scores (3 + 3, 3 + 4, and 4 + 3).

## 2. Materials and Methods

A total of 44 clinically localized PCa patients who underwent radical retropubic prostatectomy between January, 2007 and May, 2008 were selected for this study. The entire protocol was approved by the institutional review board (IRB), and an informed consent was obtained from each human subject. The ages of the patients ranged from 47 to 75 years, and the patients fell into three different groups by surgery GS: 3 + 3 (mean ± SD, 60.1 ± 6.7 years), 3 + 4 (mean ± SD, 58.1 ± 4.2 years), and 4 + 3 (mean ± SD, 60.3 ± 3.9 years). The mean prostate-specific antigen (PSA) value for the patients with GS 3 + 3, GS 3 + 4, and GS 4 + 3, respectively, were 5.0 ng/mL, 6.8 ng/mL, and 7.4 ng/mL.

 All patients underwent prostate imaging with an endorectal coil on a 1.5 Tesla Avanto-Tim MRI scanner with high-performance gradients (Siemens Medical Solutions, Erlangen, Germany). Sequences included axial turbo spin-echo (TSE) T_2_-weighted imaging through the prostate and seminal vesicles (TR/TE, 3800/101 ms; slice thickness, 3 mm; no interslice gap; field of view (FOV), 140 mm, matrix 205 × 256, slice thickness 3 mm, interslice gap 0 mm, echo-train number 32, turbo factor 13). In addition, echo-planar diffusion-weighted sequences sensitized in three orthogonal planes (TR/TE 2000/83 ms, bandwidth 1396 Hz in the EPI frequency direction) with *b* values of 0, 50, 400 s/mm^2^ were obtained at the same slice positions as the axial T_2_-weighted images. Twelve 4-mm-thick slices with no interslice gap (27 cm FOV) with three averages provided coverage of the prostate with an image acquisition time of less than a minute. Isotropic ADC maps were generated with the system software using all *b* values and taking an average value for the two directions of diffusion sensitization.

 MR Images were initially reviewed without clinical information, but the final report was generated after the clinical information was reviewed. The histology was reviewed by an experienced pathologist. The edge and the contour characteristics of the lesions were defined using the same slices on which regions-of-interest (ROI) analyses were performed. ROIs were drawn independently on the ADC maps, and differences in measurement were resolved by consensus. ROIs were drawn in the tumor PZ of all the 44 PCa patients. ADCs were calculated for all slices by

(1) $$\text{ADC}=-\frac{\ln\left(S_{1}-S_{0}\right)}{\left(b_{1}-b_{0}\right)}\;{\text{mm}}^{2}/\text{s},$$

where *S*_1_ is the signal intensity of a voxel after application of a diffusion gradient and *S*_0_ is the echo magnitude without diffusion gradients applied (*b* = 0 s/mm^2^). Diffusion sensitivity is determined by the difference between *b*_1_ and *b*_0_. If multiple tumors were present in the peripheral zone, the average ADC value was recorded for each lesion. The MRI sections and histological slices were matched on the basis of the sextant level, anterior/posterior, and peripheral/central (transitional).

 At the time of these examinations, other sequences performed as part of the routine prostate MRI protocol at our institution but not assessed in this study included sagittal and coronal TSE T_2_-weighted imaging sequences through the prostate and seminal vesicles.

## 3. Statistical Analysis

Statistical analyses were performed to assess the statistical differences between ADC values for the three different Gleason scores (GS 3 + 3, GS 3 + 4, and GS 4 + 3) using one-way analysis of variance (ANOVA) with SPSS software package assuming parameters were normally distributed. A *P* value of less than 0.05 was considered to indicate a statistically significant difference. To explore for any relationship between the ADC value, tumor volumes, and the Gleason score, Pearson correlation was performed on the data. Also, analysis of covariance (ANCOVA) was done on ADC values of different Gleason scores with tumor volume as a covariate to see its effect.

## 4. Results

**Figure 1:**
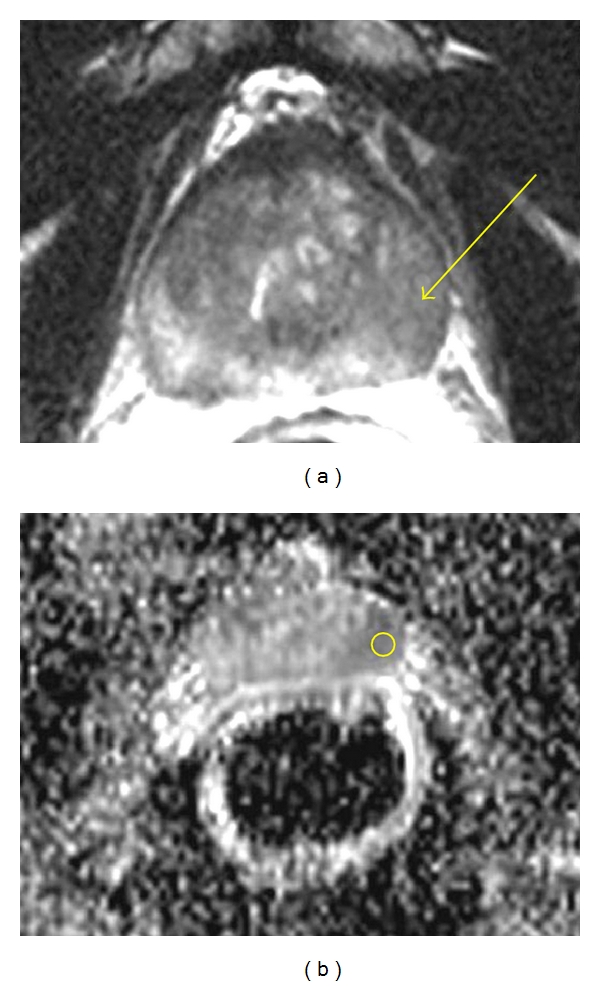
(a) T_2_-weighted MRI of 68 yo prostate cancer patient with GS 3 + 4 and (b) corresponding ADC map with low signal on the left base PZ.

**Figure 2:**
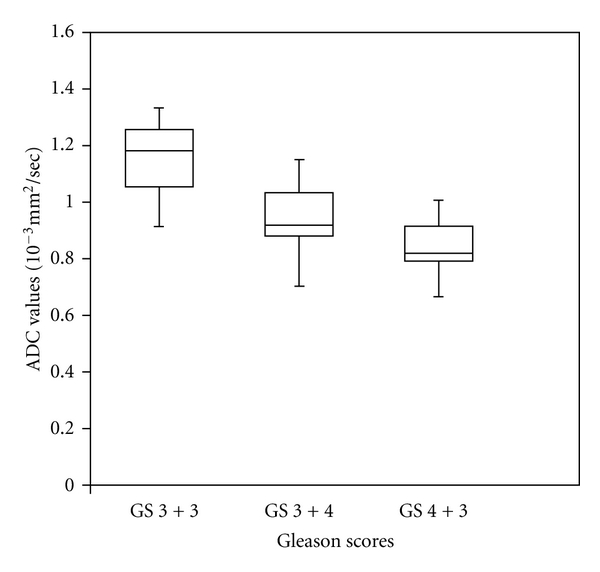
Box-Whisker plots of ADC values of Gleason score (GS) 3 + 3, (GS) 3 + 4 and (GS) 4 + 3 patients. The center horizontal line indicates the median.

The patients mean and standard deviation (SD) of age and PSA and ADC values for tumor PZ regions of three Gleason scores are shown in Table 1.  Figure 1(a) shows the T_2_-weighted MRI of a 68-year-old PCa patient with GS 3 + 4 and Figure 1(b), corresponding ADC map with low signal on the left base PZ.  Figure 2 illustrates a box plot of ADC values for PCa in the peripheral zone tissue categorized by the three Gleason scores. In 13 patients with GS 3 + 3, the (mean ± SD) ADC value was 1.135 ± 0.119 mm^2^/sec using 32 ROIs. In 22 patients with GS 3 + 4, the (mean ± SD) ADC value was 0.976 ± 0.103 mm^2^/sec using 52 ROIs. In 9 patients with GS 4 + 3, the (mean ± SD) ADC value was 0.831 ± 0.087 mm^2^/sec using 24 ROIs. Although a statistically significant difference existed between the three groups (*P* < 0.05), a certain degree of overlap between tissue types was evident. There was no statistical significance between the PSA and patients ages with three different Gleason scores.

We did not have the biopsy report for 14 patients out of 44 patients. Out of which 15 patients whose biopsy and prostatectomy Gleason scores were the same. For the remaining 15 patients, We had four patients with GS 3 + 3, eight patients with GS 3 + 4, and 3 patients with GS 4 + 3 whose biopsy Gleason scores were different from prostatectomy. Hence, we have not done the correlation between biopsy and prostatectomy Gleason scores. Out of 44 patients, in 35 patients (GS 3 + 3 (*n* = 13), GS 3 + 4 (*n* = 13), and GS 4 + 3 (*n* = 9)) tumors were detected by the DWI technique. Nine patients (GS 3 + 4 (*n* = 9)) were missed by the MRI technique. To evaluate the association between ADC value, tumor volumes, and the Gleason grade, all the ADC values and tumor volumes were first summarized at the individual patient level, followed by applying Pearson's correlation coefficients. We observed negative correlation between Gleason score and ADC values and positive correlation between Gleason score and tumor volume. In the ANCOVA analysis, the results were statistically significant (*P* = 0.0001) between the Gleason score and ADC values.

## 5. Discussion

To increase the accuracy of MRI, a number of authors have used special techniques to study a particular characteristic of the prostate tumor and surrounding tissues such as dynamic contrast-enhanced (DCE) MRI [17–19] and MR spectroscopy (MRS) [20–24]. MR spectroscopy requires a substantially longer examination time than DWI, and, additionally, shimming process and placement of saturation bands during the examination are time consuming. For evaluation of MRS, baseline correction and phase correction have to be performed in some cases.

 DWI is the only functional imaging technique that is able to assess molecular diffusion in vivo and provides information about biophysical properties of tissues such as cell organization, density, and microstructure [25]. DWI may be helpful in differentiating high-risk patients from those at low and intermediate risks, since there is a significant correlation between the ADC values from patients with three different Gleason scores. The patients with the Gleason score of 4 + 3 have a higher likelihood of biochemical recurrence, particularly for the increasing proportion of patients with organ-confined disease after radical prostatectomy than those with 3 + 4 as reported by Sakr et al. [26]. Also, our results showed decreased ADC values in patients with GS 4 + 3 than those with GS 3 + 4 significantly (*P* < 0.05). This may be useful to assess the aggressiveness of the PCa. The sensitivity of DWI is better in the PZ than the central gland [27]. DWI has also been shown to be helpful in the identification of PCa in patients with previous negative biopsies and persistently elevated PSA [28].

McNeal et al. [29] reported that 65% of prostate cancers arise in the PZ and up to 30% arise in the transition zone (TZ). The transrectal MRI is generally considered less specific for use in the evaluation of TZ cancers because of the heterogeneously low T_2_ signal intensity in normal TZ [30], and the presence of benign prostatic hyperplasia in TZ [31, 32]. The endorectal coil offers poor signal sensitivity when it comes to the TZ. Hosseinzadeh and Schwarz [33] successfully investigated T_2_ relaxation rates and diffusion-weighted images of the human prostate using an endorectal coil. DWI can provide valuable cellular information about tissue in addition to the conventional T_1_ and T_2_-weighted imaging [34–38]. In this present study, ADC values show a decreasing trend with increasing Gleason scores. The calculated ADCs for cancer in the PZ were consistent with those previously reported studies [25, 39–41]. These findings suggest that measurement of ADC may provide an additional feature that could further increase the specificity of diagnosis for PCa. The variation in reported ADC values could be due to a number of physiologic factors (e.g., age tumor size) as well as technical factors (e.g., variations in acquisition parameters, inhomogeneous signal reception using the endorectal MRI coil, and postprocessing methods).

 Our study suffered some limitations. We analyzed cancer only in the PZ, where most cancers occur. DWI itself also has some limitations as this sequence is affected by magnetic susceptibility, resulting in spatial distortion and signal loss. Moreover, there is no consensus on the optimal *b* value for DWI of the prostate.

 In conclusion, this study shows that the DWI correlates with pathological Gleason scores. DWI-acquired ADC values are a very potential measure to delineate prostate carcinoma from the PZ and are able to predict the presence of low, and high-grade components in PCa with great accuracy. The ADC values derived from the 1.5 T diffusion-weighted MRI demonstrate tumor aggressiveness and could be of future use in treatment decisions and in patient followup in active surveillance.

## Figures and Tables

**Table 1:** Patient clinical information and ADC values.

Gleason scores	Age mean ± SD	PSA mean ± SD (ng/mL)	ADC values mean ± SD (mm^2^/sec)
3 + 3 (*n* = 13)	60.1 ± 6.7	5.0 ± 2.8	1.135 ± 0.119
3 + 4 (*n* = 22)	58.1 ± 4.2	6.8 ± 1.7	0.976 ± 0.103
4 + 3 (*n* = 9)	60.3 ± 3.9	7.4 ± 2.8	0.831 ± 0.087
*P* value	NS	NS	<0.05***

NS—Nonsignificant

***—Significant.
